# Structural Prediction and Antigenic Characterization of Recombinant Nucleocapsid Protein (p14) of Small Ruminant Lentivirus

**DOI:** 10.3390/v18070803

**Published:** 2026-07-21

**Authors:** María Azucena Castañeda-Montes, José Luis Cerriteño-Sánchez, Julieta Sandra Cuevas-Romero, Francisco Jesus Castañeda-Montes, Dalia González-Esparragoza, Lucero de María Ávila-De la Vega, Hugo Ramírez-Álvarez

**Affiliations:** 1 Laboratorio de Virología, Genética y Biología Molecular, Facultad de Estudios Superiores, Cuautitlán, Medicina Veterinaria, Campo 4, Universidad Nacional Autónoma de México, Km 2.5 Carretera Cuautitlán-Teoloyucan San Sebastián Xhala, Cuautitlán Izcalli C.P. 54714, Mexico; azucena.castaneda.montes@gmail.com (M.A.C.-M.); luceroanimal@hotmail.com (L.d.M.Á.-D.l.V.); 2 Dirección de Procesos Alimentarios y Química Área Biotecnología, Unidad Académica de Capulhuac, Universidad Tecnológica del Valle de Toluca, Lerma C.P. 52700, Mexico; 3 Centro Nacional de Investigación Disciplinaria en Salud Animal e Inocuidad (CENID-SAI), Instituto Nacional de Investigaciones Forestales, Agrícolas y Pecuarias (INIFAP), KM. 15.5 Carretera México-Toluca, Col. Palo Alto, Cuajimalpa, México City C.P. 05110, Mexico; cerriteno.jose@inifap.gob.mx; 4 División de Ciencias de la Salud, Biológicas y Ambientales (DSCBA), Universidad Abierta y a Distancia de México (UnADM), Av. Universidad 1200, Piso 1 Cuadrante 1-21, Col. Xoco, Alcaldía Benito Juárez, México City C.P. 03330, Mexico; fjcastmont@gmail.com; 5 Laboratorio de Microbiología y Virología de las Enfermedades Respiratorias del Cerdo, Facultad de Estudios Superiores, Cuautitlán, Medicina Veterinaria, Campo 4, Universidad Nacional Autónoma de México, Km 2.5 Carretera Cuautitlán-Teoloyucan San Sebastián Xhala, Cuautitlán Izcalli C.P. 54714, Mexico; daly.qfb04@gmail.com

**Keywords:** antigenicity, nucleocapsid, p14, retroviruses, SRLV, molecular docking, lentiviruses

## Abstract

Small ruminant lentivirus (SRLV) infects goats and sheep of all breeds and ages worldwide. There are no current records regarding the three-dimensional structure or antigenic capacity of the nucleocapsid protein p14 of FESC-752 Mexican strain. The antigenic structure of p14 protein of a B1 genotype was predicted. cDNA from FESC-752 was used to overexpress the recombinant SRLV-rp14 protein. Then, its antigenicity was verified in vitro by evaluating plasma samples from goats and sheep naturally infected with SRLV. Antigenicity prediction showed a “horseshoe”-type structure shared by different lentiviruses and five epitopes distributed throughout the p14 surface regions where they coincide suggesting conserved epitopes in the zinc-finger structures of the nucleoproteins of the SRLV, Human Immunodeficiency Virus (HIV-1), and Feline Immunodeficiency virus (FIV) retroviruses. Multi-species molecular docking showed a notable structural convergence where caprine, bovine, murine, and human immunoglobulins target a predictive 23 amino acid epitope (residues 41–64) within the core zinc-finger region. Furthermore, CABS docking simulations predicted that p14-derived peptides preferentially bind within the antigen-presenting cleft of both caprine and bovine major histocompatibility complex class I (MHC-I) molecules. The stability of these immunological complexes is mediated by dense networks of hydrophobic interactions and highly conserved aromatic anchoring residues. Antigenicity analysis revealed that 78.7% of samples from naturally infected goats showed immunoreactivity toward SRLV-rp14 and the predictive evidence that p14 can simultaneously stimulate both humoral and cellular pathways makes it a strategic candidate for the design of next-generation vaccines aimed at controlling lentiviruses in small ruminants.

## 1. Introduction

Lentiviruses share common characteristics regarding their virion structure, genetic organization, infection kinetics, tropism, stimulated immune response, transmission, etc. [1]. Small ruminant lentivirus (SRLV) infects goats and sheep of all breeds and ages worldwide, causing a syndrome characterized by interstitial pneumonia, mastitis, encephalitis, lymphadenopathy, arthritis, and weight loss, resulting in significant economic losses [2]. SRLV is a ribonucleic acid virus, enveloped by a membrane, belonging to the Ortervirales order, Retroviridae family, Orthoretrovirinae subfamily, and genus Lentivirus [3]. Its genome is composed of two single-stranded subunits in the positive sense that measure between 8.4 and 9.2 Kb, and its genetic organization includes three main genes, *gag*, *pol*, and *env*, and three accessory genes, *vif*, *vpr-like*, and *rev* [4,5]. The *gag* genes encode nucleocapsid (NC), capsid (CA), and matrix (MA) proteins; the *pol* genes encode reverse transcriptase, protease, integrase, and dUTPase enzymes; and the *env* genes encode transmembrane (TM) proteins and surface (SU) glycoproteins [6]. The protein products of *gag* and *env* genes contain antigenic regions. An immunoreactivity pattern toward the amino terminus of the p25 protein and the carboxyl terminus of the p14 protein and an antibody response toward Gag proteins p14, p16 and p25 have been observed [7,8,9]. Recently, the antigenicity of the p25 recombinant protein (SRLV-rp25) of the SRLV genotype B1 strain FESC-752 was demonstrated using plasma from naturally infected sheep and goats from different states of Mexico [10]. While the p25 capsid protein has been extensively investigated for serodiagnosis and subunit development, the nucleocapsid protein p14 remains largely neglected in regional molecular epidemiology and structural biology frameworks [11,12,13]. Crucially, there are currently no available records regarding the three-dimensional structure, spatial epitope distribution, or in vitro antigenic capacity of the p14 nucleocapsid protein belonging to Mexican field strains. Given that the retroviral nucleocapsid domain alternates between functional structural conformations required for viral genomic RNA binding, dimerization, and encapsidation [14,15,16], uncovering its baseline immunology is critical to identifying target sites for multi-epitope diagnostics or modern vaccine formulations.

In modern virology, structural biology approaches, specifically molecular docking, have emerged as crucial in silico computational tools to bridge the gap between static genetic sequences and dynamic functional immunology [17,18]. To simulate the complexity of host–pathogen interactions, docking protocols can be executed under rigid or flexible parameters [19,20]. Rigid docking considers both interacting structures as static entities, which is highly efficient for large multimeric surfaces but underrepresents the natural “induced fit” phenomenon that occurs during biological recognition [21,22]. Conversely, flexible docking, such as that executed by the CABS-dock platform, implements continuous conformational sampling [23,24]. This allows the ligand and receptor side chains to dynamically adjust their shapes, which is a methodological requirement when evaluating small, highly flexible antigenic peptides that amorphously accommodate within constrained binding pockets [25]. Furthermore, executing comparative multi-species docking protocols enables the assessment of cross-reactive structural conservation, identifying whether disparate host immunoglobulin lineages converge toward identical surface regions (*hotspots*) based on electrostatic charges and solvent accessibility, independent of host-specific sequence variations [26,27].

Therefore, in this work, the structure of the p14 protein of SRLV of the FESC-752 strain was predicted, and the antigenicity of the recombinant protein SRLV-rp14 was determined, as well as plasma samples from naturally infected goats and sheep from various states of Mexico. Furthermore, to elucidate the mechanistic basis of host immune recognition, comparative multi-species molecular docking was performed against caprine, bovine, murine, and human immunoglobulins, complemented by flexible peptide–protein docking (CABS-dock) to simulate how p14-derived antigenic determinants are processed and presented by host major histocompatibility complex class I (MHC-I) molecules.

## 2. Materials and Methods

### 2.1. Structural and Antigenic Prediction of p14 Protein via In Silico Analysis

The coding sequence of the p14 protein reported in GenBank with accession number HM210570.1 from a Mexican genotype B1 isolate was used. The DNAStar program (DNASTAR Inc., Madison, WI, USA) was used to determine the hydropathicity index with the “Kyte and Doolittle” algorithm, the antigenic index with the “Jameson–Wolf” algorithm, and the surface probability index with the “Emini” algorithm [28,29,30]. Nucleoprotein sequences from different lentiviruses were selected using the NCBI protein-BLAST server (National Center for Biotechnology Information, National Library of Medicine, Bethesda, MD, USA) [31], and the following sequences were obtained: EIAV (NP_056901.1), FIV (ABO16618.1), and HIV-1 (AUO17779.1). The epitope distribution determination of these sequencies was performed using the Ellipro server (Immune Epitope Database and Analysis Resource, La Jolla Institute for Immunology, La Jolla, CA, USA) to identify antigenic determinants [32]. Epitope distribution modeling was performed using the I-TASER server (Zhang Lab, Department of Computational Medicine and Bioinformatics, University of Michigan, Ann Arbor, MI, USA) [33], and the prediction of tertiary structures was obtained using the PyMOL Molecular Graphics System v.2.0 (Schrödinger, LLC, New York, NY, USA) [34].

### 2.2. Biological Material

Previously, the template cDNA was obtained and used to amplify the ORF of the p14 protein (GenBank accession number HM210570.1). This cDNA was donated by Dr. Hugo Ramírez from FESC-UNAM and synthesized from the supernatant of synovial membrane cells from a goat experimentally infected with the viral strain FESC-752 that had previously been isolated and identified in Mexico [35]. The amplified open reading frame (ORF) of the p14 protein was cloned into the Champion pET-SUMO expression plasmid (Thermo Fisher Scientific, Waltham, MA, USA) and transformed into *E. coli* strain One Shot™ BL21 (DE3) (Thermo Fisher Scientific, Waltham, MA, USA).

### 2.3. PCR Amplification and Cloning of ORF of SRLV-rp14 Protein

The oligonucleotides foward-p14 (5′-TTG TTA GCA CAA GCC TTA AGG CC-3′) and reverse-p14 (5′-CTA CTA TTC CAT AGG AGG AGC GGA C-3′) were used to amplify the p14 protein coding sequence using PCR. These oligonucleotides were hybridized within the ORF of the SRLV p14 gene of genotype B1 and designed to be able to clone the sequence in the reading frame with the N-terminal end of the Champion pET-SUMO expression vector. This system incorporates a 6xHis-SUMO tag at the N-terminal to improve solubility, detection, and purification of recombinant protein. This tag increases the protein molecular weight by approximately 13 kDa [36]. The reaction mixture volume for PCR was 50 μL, containing 25 μL of 2× Master Mix (Thermo Fisher Scientific, Waltham, MA, USA), 1 μM of each primer, and 1 μg of the template cDNA. The PCR products were analyzed via 1.5% (*w*/*v*) agarose gel electrophoresis. The amplified 246 bp fragment was purified from the gel using the QIAquick^®^ Gel extraction kit (QIAGEN, Hilden, Germany) and then inserted into the pETSUMO expression vector to obtain the pETSUMO-SRLV-rp14 plasmid. The *E. coli* Top10 strain (Thermo Fisher Scientific, Waltham, MA, USA) was transformed with the pETSUMO-SRLV-rp14 vector, and colonies were selected in LB medium with 50 μg/mL of kanamycin. Recombinant plasmid extraction was performed using the QIAprep Spin^®^ Miniprep kit (QIAGEN, Hilden, Germany) according to the manufacturer’s instructions. The correct orientation of the insert inside the expression vector was confirmed using PCR, restriction enzymes, and Sanger sequencing.

### 2.4. Expression and Affinity Column Purification of SRLV-rp14 Recombinant Protein

BL21 (DE3) cells competent with MgCl_2_ and CaCl_2_ were transformed with 50 ng of pETSUMO-SRLV-rp14 vector, which contained the open reading frame of the p14 protein via heat shock at 42 °C for 90 s. Transformants were selected on Luria–Bertani (LB) (BD Difco™, Becton, Dickinson and Company, Sparks, MD, USA) agar plates containing 50 µg/mL of kanamycin (Sigma-Aldrich, Burlington, MA, USA. The selected clones were named BL21-SRLV-rp14. Then, a pre-culture was performed in 10 mL of LB–kanamycin (50 µg/mL) inoculated with the BL21-p14 clone and incubated at 37 °C and 250 rpm for 12 h. A larger culture of 100 mL of the LB–kanamycin medium (50 µg/mL) synchronized at an optical density (OD) of 0.1 units at 600 nm was inoculated with this and subsequently incubated at 37 °C and 250 rpm until it reached an OD of 0.5 units. At this point, protein expression was induced with 1 mM of isopropyl β-D-1-thiogalactopyranoside (IPTG) (Merck KGaA, Darmstadt, Germany). After 12 h of induction, protein expression was analyzed using SDS-PAGE and Western blot [10]. Cells from the induced medium were centrifuged at 5000 rpm for 10 min at 4 °C to recover the recombinant protein. The cell pellet was washed with 10 mL of distilled H_2_O and centrifuged at 5000 rpm for 10 min at 4 °C. Next, the mechanically broken pellet was obtained using a GAULIN homogenizer (Gaulin APV Homogenizer Group, Wilmington, MA, USA) at 540 kg/cm^2^ for 15 min, and the precipitate was separated from the supernatant via centrifugation for 15 min at 8000 rpm for 20 min at 4 °C. Finally, the recombinant protein was solubilized from the soluble phase using a binding buffer (4.5% N-lauroylsarcosine sodium salt and 50 mM Tris-HCl (pH 8)) under constant stirring at 250 rpm for 12 h at 25 °C. Solubilized inclusion bodies were purified using a Ni-NTA agarose column (Sigma Aldrich, San Luis, MO, USA), with 5 mL of resin packed inside the vertical column, to purify the recombinant SRLV-rp14 [10]. Absorbance at 280 nm was measured to obtain the elution profile. The presence of the purified viral protein was confirmed using SDS-PAGE and Western blot. Finally, the protein was dialyzed, lyophilized, and stored at −80 °C until use. Subsequently, it was resuspended, and the concentration was measured via the Bradford method using bovine serum albumin as a standard [37].

### 2.5. Immunodetection of Recombinant Protein SRLV-rp14

A specific conjugated antibody directed toward the histidine tag incorporated from the plasmid was used for the immunodetection of the recombinant protein. Briefly, 300 ng of the recombinant protein was separated using SDS-PAGE and transferred onto a 0.45 µm nitrocellulose membrane (BioRad, Hercules, CA, USA) and blocked in a blocking solution (1× PBS pH 7.4, 0.1% Tween 20, and 5% fat-free milk) for 1 h under constant stirring. For the protein detection, the membrane was washed with buffer (1× PBS pH 7.4 and 0.1% Tween 20) and incubated with anti-his antibodies (Fine Test, Boulder, CO, USA) diluted to 1:10,000 in the blocking solution. Next, three washes were performed with the washing solution (1× PBS pH 7.4 and 0.1% Tween 20), and it was incubated for two h with mouse anti-IgG conjugated to horseradish peroxidase produced in rabbit (dilution 1:5000) (Sigma Aldrich, San Luis, MO, USA) diluted to 1:5000 in the blocking solution. After three washes with the washing solution, the protein bands were revealed with a DAB solution (1× PBS pH 7.4, 12 mg of 3,3′-diaminobenzidine tetrahydrochloride, and 300 µL of 3.4% H_2_O_2_). Expasy-ProtParam (Swiss Institute of Bioinformatics, Lausanne, Switzerland) was used to determine the estimated half-life of recombinant nucleoprotein p14 [38].

### 2.6. Antigenicity Evaluation of Recombinant Protein SRLV-rp14 Toward Plasma from Infected Goats and Sheep by Western Blot

Plasma samples from naturally infected goats and sheep were donated by Dr. Hugo Ramírez from FESC-UNAM and reported by Mendiola and collaborators in 2019 [39]. These samples were used in this analysis to determine the antigenic characteristics of the recombinant viral protein expressed in a heterologous system. These samples corresponded to plasma of female and male sheep and goats from several states of Mexico where the disease is endemic: Durango, Chiapas, Hidalgo, Sinaloa, Coahuila, Guanajuato, Querétaro, Tlaxcala, Baja California Sur, Sonora, the State of Mexico, and Veracruz (Table A1, Appendix A). The samples had previously been assessed using commercial kits VMRD CAEV/MVV Antibody Test Kit (VMRD Inc., Pullman, WA, USA)**,** based on competitive ELISA directed to gp135, and ERADIKIT™ SRLV ELISA kit (In3diagnostic, Turin, Italy),based on indirect ELISA directed to gag peptides, and the age range of the animals was 1–10 years [39]. Some sheep had respiratory disease, and some goats had arthritis and mastitis. Western blot assays were performed using 300 ng of the SRLV-rp14 protein for the immunoreactivity detection of the plasmas from the infected animals. Each plasma was diluted to 1:1000, and peroxidase-conjugated recombinant protein G (Thermo Fisher Scientific, Waltham, MA, USA) was used as a secondary antibody, diluted to 1:5000 in a blocking solution. The protein bands were observed with 10 mL of a DAB solution. The antigenicity degree was categorized by comparing the intensity per pixel between the positive control and serum samples using the ImageJ program version 1.54p (National Institutes of Health, Bethesda, MD, USA). The signal intensity of the positive control was considered the highest degree of immunoreactivity (++++), and the signal intensity of the analyzed samples was compared to this using densitometric analysis with ImageJ. Thus, a high degree had four recognition marks (++++), a moderate degree had three (+++), a medium degree had two (++), and a low degree had one (+); the negative control serum was designated with a mark (-) [40].

### 2.7. Three-Dimensional Modeling of Caprine Immunological Macromolecules Using AlphaFold Server

Because crystallographic structures for goat and sheep immunoglobulins and major histocompatibility complex class I (MHC-I) molecules are unavailable in the PDB, we used computational in silico modeling to predict the 3D structures of these macromolecules for host-specific modeling. Amino acid sequences corresponding to the variable region of the heavy chain of the *Capra hircus* antibody (GenBank accession: AAX45026.1), the variable region of the light chain (GenBank accession: AAX45027.1), and the caprine MHC-I molecule (UniProt accession: A5HTU8) were retrieved from the NCBI and UniProt databases, respectively. Three-dimensional structural models were generated using the AlphaFold server platform [41], which employs deep learning algorithms that integrate evolutionary information derived from multiple sequence alignments and coevolutionary patterns to predict protein conformations. Multiple structural predictions were generated for each sequence, and the final model was selected according to AlphaFold (Google DeepMind, London, UK, and Isomorphic Labs, London, UK) confidence metrics, prioritizing the overall predicted structural quality and the distribution of highly reliable regions. Model confidence was assessed using the predicted Local Distance Difference Test (pLDDT), predicted Template Modeling score (pTM), and interface predicted Template Modeling score (ipTM). The pLDDT provides a residue-level confidence estimate on a scale from 0 to 100, with higher values indicating greater reliability of local structural predictions. The pTM score evaluates the accuracy of the overall protein fold, with values above 0.5 generally indicating a reliable global conformation. For multimeric complexes, the ipTM score was used to estimate the accuracy of relative subunit positioning, with values above 0.8 considered indicative of high-confidence interface predictions. The structural quality of the generated models was assessed through stereochemical parameters, overall folding analysis, and evaluation of backbone dihedral angle distributions. Structural assessment was performed using Ramachandran plots generated with the Ramplot server (Indian Institute of Science Education and Research, Bhopal, Madhya Pradesh, India) [42]. Models were considered suitable for subsequent molecular docking analyses when more than 95% of residues were located within favored regions.

### 2.8. Comparative Multispecies Humoral Recognition Profiling Through Multimeric Docking Using AlphaFold

The AlphaFold generated caprine antibody model was evaluated in a comparative framework with experimentally determined mammalian immunoglobulin structures available in the PDB database, including the ultralong bovine antibody BOV-2 (PDB ID: 6E9G) [43], the murine antibody 1EJO (PDB ID: 1EJO) [44], and the human antibody 5I17 (PDB ID: 5I17) [45]. These structures were used as receptor molecules for comparative protein–protein interaction analyses. The complete three-dimensional structure of the SRLV p14 nucleocapsid protein from strain FESC-752 was used as the interacting ligand. Structural alignments and sequence identity percentages between the heavy and light antibody chains were determined using NCBI BLAST to evaluate molecular conservation among species. Protein-protein interfaces were characterized by analyzing hydrogen bonds and hydrophobic contacts between antibody and antigen molecules. Intermolecular interactions were identified using LigPlot implemented in the LigPlus package version 2.3.1 (European Bioinformatics Institute, Hinxton, Cambridge, UK) [46,47] and subsequently visualized and analyzed using Maestro v14.6 (Maestro, Schrödinger, LLC, New York, NY, USA, 2025) [48].

### 2.9. Modeling of Antigenic Peptide Presentation by MHC-I Molecules Using CABS-Dock

The potential interaction between antigenic determinants derived from the SRLV p14 protein and caprine MHC-I molecules was evaluated through flexible peptide–protein docking simulations using the CABS-dock platform (CABS Laboratory, Faculty of Chemistry, University of Warsaw, Warsaw, Poland) [49]. This approach enables simultaneous conformational sampling of both ligand and receptor molecules during simulation, considering peptide flexibility and receptor adaptation without requiring prior definition of a binding site [50]. The five previously predicted linear epitopes from the SRLV p14 protein, Pep1 (RPERKKGPGQ), Pep2 (GKPGH), Pep3 (NCGKRGHMQKDCSKRDMRGKQQG), Pep4 (RRGIR), and Pep5 (SAPPME), were used as peptide ligands to evaluate their interaction with the antigen-binding groove of the caprine MHC-I molecule modeled using AlphaFold server. Additional docking simulations were performed using the experimentally determined structure of the bovine MHC-I molecule BoLA-A11 (PDB ID: 3PWV) [51] to evaluate the conservation of peptide-binding patterns among ruminant species. The resulting peptide–MHC complexes were hierarchically grouped into 10 clusters by the CABS-dock server, and representative conformations were selected based on cluster density. Subsequently, intermolecular interactions, including hydrogen bonds and hydrophobic contacts between peptides and MHC-I molecules, were identified using LigPlot and DimPlot implemented in the LigPlus package version 2.3.1 and further analyzed using Maestro v14.6.

## 3. Results

### 3.1. Structural Prediction and Antigenic Determinants of Nucleocapsid Proteins of the Lentiviruses SRLV, HIV-1, FIH, and EIAV

**Figure 1:**
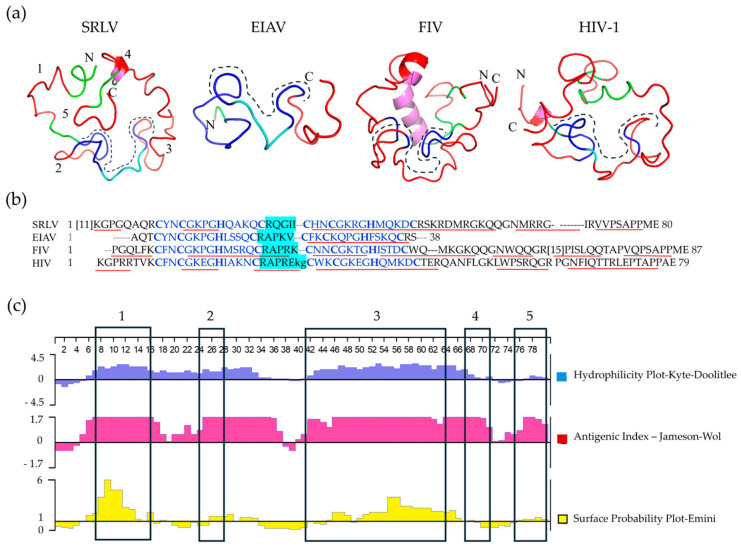
Antigenic and structural comparison of nucleocapsid proteins from different Lentiviruses. (**a**) Comparison of three-dimensional epitope distribution models of nucleocapsid proteins of the Lentivirus SRLV (HM210570.1), Equine Infectious Anemia Virus EIAV (NP_056901.1), Feline Immunodeficiency Virus FIV (ABO16618.1), and Human Immunodeficiency Virus HIV-I (AUO17779.1). Numbers indicate epitopes found within the structure. Antigenic determinants obtained with the Ellipro server are in red, zinc fingers are indicated in blue, zinc finger binding sequences are marked in turquoise, Alpha-helices are marked in lilac, dashed black line indicates the “horseshoe” shape characteristic of zinc fingers. (**b**) Amino acid sequence for each predictive model, sequences in blue correspond to the zinc fingers of each of the analyzed proteins, antigenic sites are underlined in red, the zinc finger binding sequence is highlighted in turquoise. (**c**) Surface properties of the SRLV sequence analyzed, the hydrophilicity, antigenic index and surface probability are observed, black rectangles indicate the antigenic determinants obtained through the Ellipro site.

A structural and antigenic comparison of the nucleocapsid proteins of SRLV, human immunodeficiency virus type 1 (HIV-1), feline immunodeficiency virus (FIV), and equine infectious anemia virus (EIAV) was performed to determine the similarity of nucleocapsid protein p14 among the lentiviruses. The amino acid sequences with greater similarity to p14 were selected by analyzing the p14 protein sequence in the NCBI protein-BLAST server. The nucleoprotein sequences with the highest similarity were EIAV (NP_056901.1), FIV (ABO16618.1), and HIV-1 (AUO17779.1), with identity percentages of 59.87%, 43.33%, and 41.98%, respectively. The I-TASER platform and the PyMOL program were used for the three-dimensional modeling of the nucleocapsid sequences obtained. A comparison of the models showed that more than 40% is enough to high in three-dimensional modeling homology between the proteins, and they shared a “horseshoe”-type structure corresponding to the zinc fingers characteristic of proteins that bind nucleic acids such as nucleoproteins (Figure 1a).

The predicted epitopes obtained by Ellipro server were predominantly located within the sequence and tridimensional zinc-finger structure. In the p14 protein of SRLV, the zinc-finger structure had the segment 20-CYNCGKPGHQAKQC-33 followed by the binding sequence 34-RQGII-38 and then the segment 39-CHNCGKRGHMQKDC-52. In the EIAV nucleocapsid protein, this structure was composed of the segment 4-CYNCGKPGHLSSQC-17 followed by the binding sequence 18-RAPKV-22 and then the segment 23-CFKCKQPGHFSKQC-36. In the FIV nucleocapsid protein, the structure consisted of the segment 7-CFNCGKPGHMSRQC-20 followed by the binding sequence 21-RAPRK-25 and then the segment 26-CNNCGKTGHISTDC-39. In the nucleocapsid protein of the HIV-1 virus, the “horseshoe” consisted of the segment 9-CFNCGKEGHIAKNC-22, the binding sequence 23-RAPREKG-29, and then the segment 30-CWKCGKEGHQMKDC-43 (Figure 1b). Likewise, in the three-dimensional model and the primary structure of the nucleoproteins, each of the epitope sequences or antigenic determinants found with the Ellipro server is indicated in red (Figure 1a,b and Table 1). Some epitopes coincided in certain regions in the three-dimensional models and the virus’s primary structures, mainly where the zinc fingers were located. However, the sequences were not conserved among the nucleoproteins of the different lentiviruses (Figure 1a,b). A conserved epitope was observed at the end of the amino acid sequences of the nucleoclapsid of SRLV and FIV (Figure 1b and Table 1). To corroborate these results, the surface properties of the SRLV p14 protein sequence were analyzed using the DNAstar program; the Kyte and Doolittle algorithms to determine the hydrophilicity; the Jameson–Wolf algorithm to indicate the antigenic index; and the Emini algorithm to obtain the surface probability (Figure 1c).

Sequence analysis of p14 protein of SRLV revealed the presence of multiple hydrophilic and antigenic regions along the protein. Epitopes predicted by the ElliPro server were predominantly located within these regions, as indicated by the black rectangles. The observed overlap between predicted epitopes and hydrophilic domains suggests the presence of several antigenic determinants distributed throughout the recombinant SRLV-p14 nucleoprotein (Figure 1c).

### 3.2. Cloning BL21-SRLV-rp14 Strain

**Figure 2:**
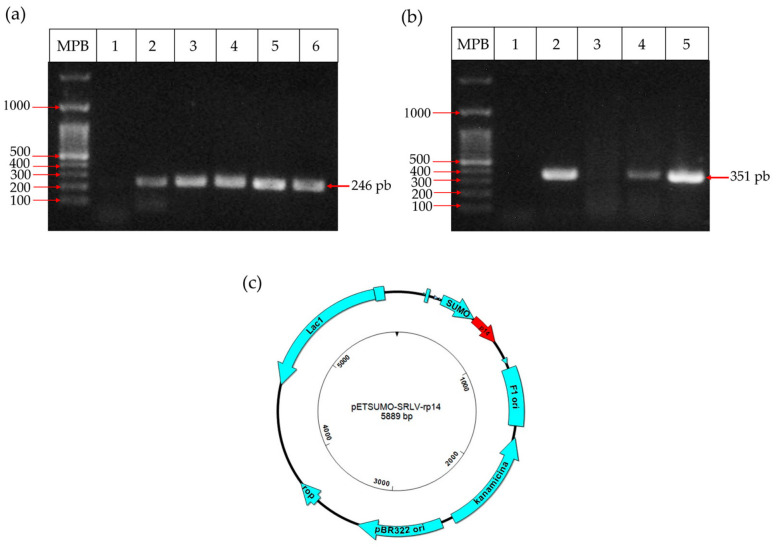
Construction of the pETSUMO-SRLV-rp14 vector. (**a**) Amplification by temperature gradient PCR of SRLV p14 protein sequence from the cDNA of experimentally infected goat with SRLV strain FESC–752; MPB corresponds to base pair marker, lane 1 is the negative control of PCR without plasmid; lanes 2–6 correspond to the temperature gradient from 50 to 60 °C. (**b**) PCR of pETSUMO-SRLV-rp14, lane 1 corresponds to negative control (no plasmid), lanes 2–5 refer to the plasmids extracted from analyzed clones. (**c**) pETSUMO-SRLV-rp14 plasmid, the location of the coding sequence of the cloned SRLV-rp14 protein is shown in red.

**Figure 3:**
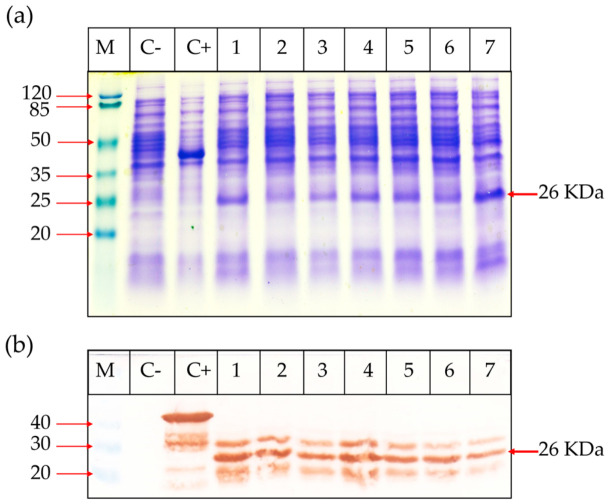
Selection of the BL21-SRLV-rp14 clone. (**a**) SDS-PAGE of the SRLV-rp14 recombinant protein expressed in different clones, (**b**) Western blot of SRLV-rp14 protein expressed in different clones. M, corresponds to the molecular weight marker; C−, negative control that corresponds to non-transformed strain; C+, BL21-CAT strain containing the pETSUMO-CAT vector was used as a positive expression control; 1anes 1–7 refers to the clones analyzed for the expression of the SRLV-rp14 recombinant protein. Different molecular weight standards were used in this figure. Panel (**a**), as well as all other gel figures presented in this study, employed the Thermo Scientific™ 26612 protein marker (Thermo Fisher Scientific, Waltham, MA, USA), whereas panel (**b**) utilized the abbexa abx098114 protein marker (Abexa, Cambridge, UK).

The p14-coding region was amplified by PCR from cDNA obtained from a goat experimentally infected with the FESC-752 strain, using a gradient annealing temperature protocol to optimize amplification efficiency and specificity. Then analyzed on agarose gels and obtained the optimal annealing temperature of 56 °C for the fragment amplification that showed an expected molecular size of 246 bp (Figure 2a). Next, this PCR product was cloned into the pETSUMO expression vector that allowed the gene to be inserted in one cloning step through the A’ end placed by Taq polymerase. Confirmation of the insert orientation demonstrated successful cloning of the SRLV-rp14 sequence, as evidenced by the amplification of a DNA fragment of the expected size from plasmids harboring the insert in the correct reading frame with the expression vector (Figure 2b). Three plasmids harboring the insert in the correct orientation were identified (Figure 2b, lanes 2, 4, and 5). The construct corresponding to lane 5 was selected for transformation into *E. coli* BL21 cells for recombinant protein expression. This expression plasmid, designated pETSUMO-SRLV-rp14, was subsequently used to produce the recombinant p14 protein. This expression system produced a recombinant protein fused to a 6-histidine-SUMO tag, which allowed for purification and improved the solubility of the expressed protein, increasing its molecular weight by approximately 13 KDa. As a result, a 26 KDa recombinant SRLV-rp14 protein was obtained (Figure 2c and Figure 3a,b).

### 3.3. Overexpression of SRLV-rp14 Recombinant Protein in E. coli

The pETSUMO-SRLV-rp14 expression vector was transformed into *Escherichia coli* BL21(DE3) cells, generating the recombinant strain BL21-SRLV-rp14. Recombinant protein expression was evaluated in seven independent transformants, and the clone exhibiting the highest expression level, as determined by comparison of overexpressed band intensities, was selected for subsequent scale-up experiments. Protein expression in the seven clones was assessed by SDS-PAGE and confirmed by Western blot analysis using anti-His antibodies.

A protein band corresponding to the expected molecular weight of approximately 26 kDa was detected, consistent with the predicted size of the recombinant SRLV-rp14 monomer described in Section 2. This result confirms the successful expression and production of the recombinant SRLV-rp14 protein. No corresponding protein bands were detected in the negative control consisting of protein extracts from non-transformed cells. As a positive control for recombinant protein overexpression, the BL21-CAT strain carrying the pETSUMO-CAT vector (Champion™ pET SUMO Expression System, Thermo Fisher Scientific, Waltham, MA, USA), which overproduces a 39 KDa chloramphenicol acetyltransferase, was included in the analysis (Figure 3a). The same samples were analyzed using Western blot with anti-his antibodies. As expected, the samples corresponding to proteins from non-transformed cells showed no signal, the CAT protein had an intense signal, and the selected colonies showed an expected signal of 26 KDa, confirming the expression of the recombinant protein (Figure 3b).

### 3.4. Purification of Recombinant Protein SRLV-rp14

**Figure 4:**
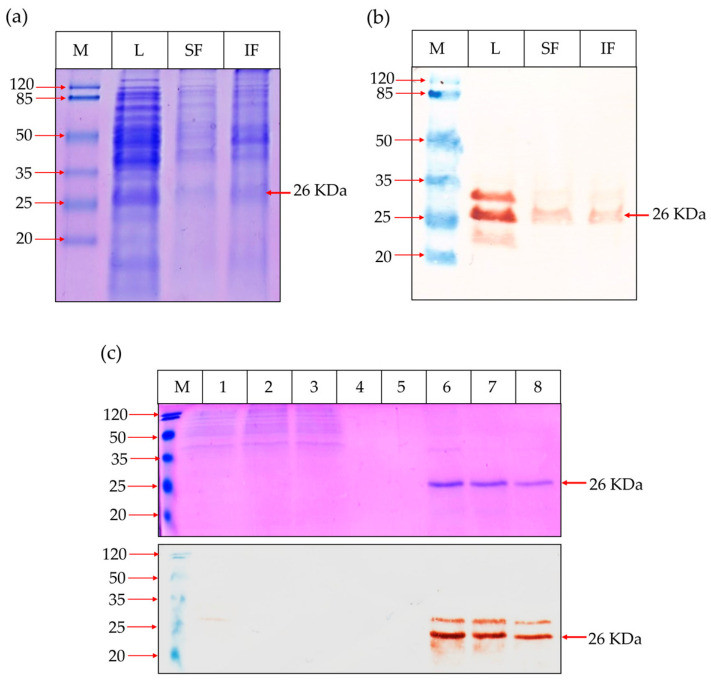
Solubility of the recombinant SRLV-rp14 protein and purification by affinity column. (**a**) SDS-PAGE electrophoresis of fractions obtained after cell disruption. M, molecular weight marker; L, total lysate obtained; SF, soluble fraction; IF, insoluble fraction. (**b**) Western blot of the fractions obtained after cell disruption. M, molecular weight marker; L, total lysate obtained; SF, soluble fraction; IF, insoluble fraction. (**c**) Purification by affinity column. M, molecular weight marker; 1, SRLV-rp14 protein solubilized with sarcosyl; 2 and 3, protein not attached to the column; 4 and 5, column washing; 6–8, elutions of SRLV-rp14 recombinant protein.

A recombinant protein can be produced in a soluble or insoluble manner; therefore, after producing the SRLV-rp14 recombinant protein in a volume of 200 mL of LB medium, the fraction in which this protein was expressed was determined to obtain an efficient yield. The cells were disrupted with a Gaulin homogenizer, and the total lysate was collected and centrifuged to separate the soluble from insoluble fractions. The samples of each fraction were then prepared, and SDS-PAGE and Western blot were performed to determine the localization in the fractions and observe the identity of the recombinant protein. The recombinant protein was detected in both soluble and insoluble fractions; however, the soluble fraction was selected for purification because it yielded a higher amount of recoverable protein and simplified the purification procedure. SDS-PAGE analysis showed a major band at the expected molecular weight, along with additional bands that may correspond to protein isoforms (Figure 4a). The soluble recombinant protein was then solubilized in sarcosyl-containing buffer and purified by Ni-NTA affinity chromatography. Following purification, bound proteins were eluted from the Ni-NTA resin using an imidazole-containing buffer. The resulting fractions were analyzed by SDS-PAGE to determine the elution profile and identify the fractions containing the recombinant protein. Protein identity was subsequently confirmed by Western blot analysis using anti-His antibodies (Figure 4b). The results of the purification process indicated no loss of the recombinant protein due to the washing carried out (Figure 4c, lanes 4 y 5). Additionally, the protein was eluted in fractions 1–3 (Figure 4c, lanes 6–8). The elutions of the recombinant were then pooled, dialyzed, lyophilized, and kept at −80 °C until use to guarantee the protein’s stability. Finally, the protein was resuspended and quantified using the Bradford method, which indicated a yield of 30 µg of the purified SRLV-rp14 protein per 100 mL of culture used. It was then used to determine the antigenicity using plasma from sheep and goats naturally infected with SRLV from different states of the country.

### 3.5. Antigenicity of SRLV-rp14 Recombinant Protein

We assessed whether the SRLV-rp14 protein, expressed in a heterologous system, could be recognized by antibodies generated after natural SRLV infection in small ruminants. Seventy-nine plasma samples obtained from animals across multiple states of an SRLV-endemic country were analyzed. Clinical signs compatible with SRLV infection were observed in a subset of animals, including respiratory disease in sheep and arthritis and mastitis in goats. The recombinant SRLV-rp14 protein displayed marked antigenic reactivity, as demonstrated by its specific recognition by antibodies detected in plasma samples from naturally infected sheep. The magnitude of the immunoreactive signal reflected the level of antigen–antibody interaction, providing evidence of the antigenic reactivity of the recombinant protein.

**Figure 5:**
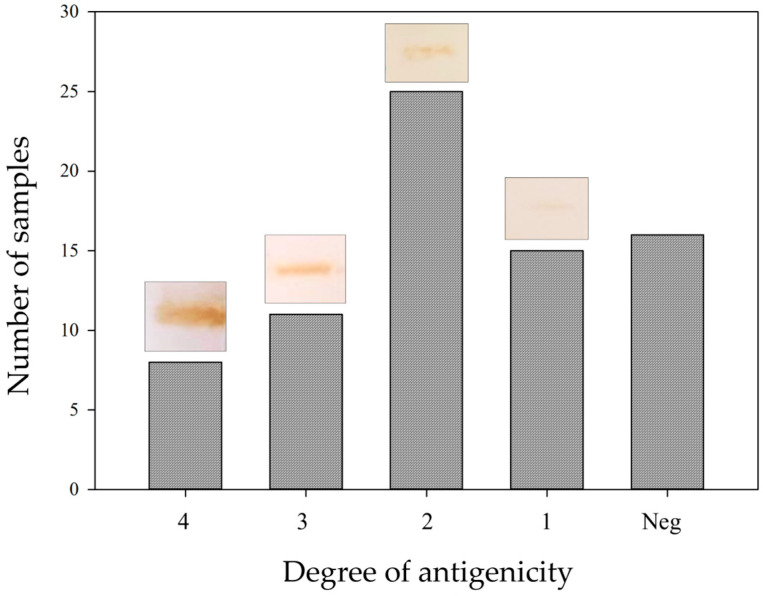
Degree of antigenicity of SRLV-rp14 protein. The *X* axis corresponds to the degree of antigenicity of the SRLV-rp14 protein by antibodies from plasma from naturally infected sheep, and the *Y* axis indicates the number of samples positive for capsid protein recognition. The signal that corresponds to each degree of antigenicity is observed.

The plasma samples analyzed in this study had been previously evaluated using commercial ELISA kits, which are routinely employed for the serological diagnosis of SRLV infection. As shown in Table A1 (Appendix A), most of the Western blot results obtained with the recombinant p14 protein were concordant with those previously generated using the commercial kits VMRD-gp135 and ERADIKIT-peptides gag. As described in Section 2, Western blot reactivity was assessed by comparing the pixel intensity of serum sample bands with that of the positive control using ImageJ software. According to this analysis, samples were categorized into four levels of immunoreactivity corresponding to signal intensity: ++++ (strong), +++ (moderate), ++ (weak), and + (faint), reflecting progressively lower antigen–antibody recognition. The results of the degree of antigenicity analysis show that 10.12% (8) of samples corresponded to recognition grade 4 (++++), 17.72% (14 samples) to recognition grade 3 (+++), 30.37% (24 samples) to recognition grade 2 (++), and 18.98% (15 samples) to recognition grade 1 (+); 22.78% (18 samples) were negative (Figure 5). These results suggest that the recombinant nucleocapsid protein SRLV-rp14 is sensitive to determining SRLV infection and can efficiently recognize samples from SRLV-infected animals.

### 3.6. Characterization of Multispecies Humoral Recognition Profiles and Convergence Toward a Central Zinc Finger Epitope

**Figure 6:**
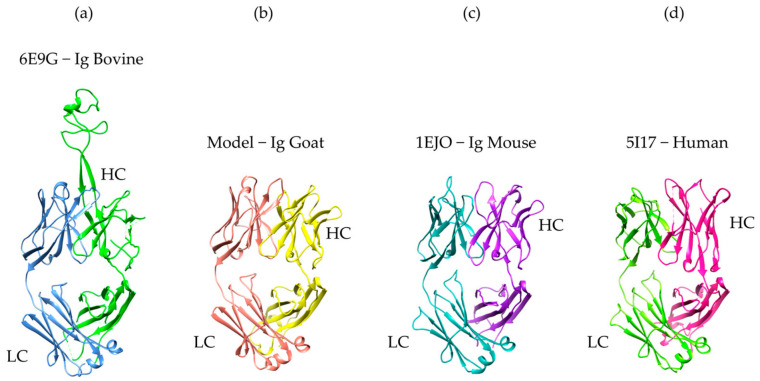
Structural characterization and comparative alignment of mammalian immunoglobulins. (**a**) Three-dimensional structure of the *Bos taurus* ultralong antibody BOV-2 (PDB ID: 6E9G). (**b**) Three-dimensional model of the *Capra hircus* antibody variable domains generated via AlphaFold server. (**c**) Crystallographic structure of the murine antibody 1EJO (PDB ID: 1EJO). (**d**) Crystallographic structure of the human antibody 5I17 (PDB ID: 5I17). HC: heavy chain; LC: light chain.

**Figure A1:**
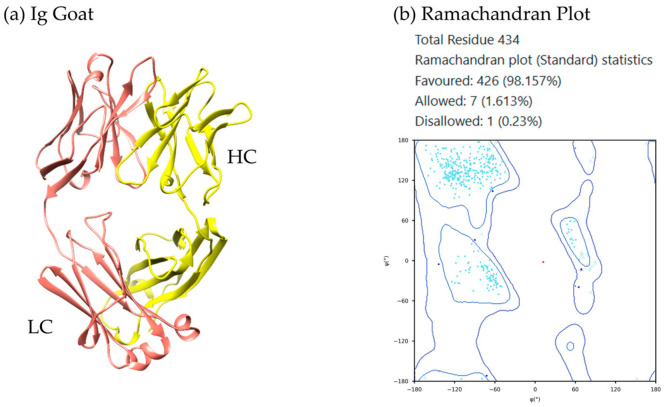
Structural validation of the AlphaFold predicted caprine immunoglobulin model. (**a**) Three-dimensional representation of the variable domains of the *Capra hircus* immunoglobulin generated using the AlphaFold server. The heavy chain (HC) and light chain (LC) domains are indicated. (**b**) Ramachandran plot analysis showing the stereochemical quality of the predicted antibody structure. The percentage of residues located in favored (light blue dots), allowed (dark blue dots), and disallowed (red dots) regions is indicated.

Despite the epidemiological and economic relevance of small ruminant lentiviruses, no crystallographic structures of ovine or caprine antibodies are currently available in the Protein Data Bank. To address this limitation and avoid potential biases associated with the exclusive use of antibody structures from heterologous species, an in silico structural modeling approach based on the AlphaFold server was employed. This deep learning-based tool enabled the accurate prediction of the three-dimensional folding of the heavy and light chains of a caprine antibody, providing a host-specific structural model required for reliable molecular docking simulations. The predicted antibody structure was assessed through Ramachandran plot analysis, showing that 98.15% of residues were located within favored regions (Figure A1). The caprine antibody model was compared to experimentally resolved bovine (6E9G), murine (1EJO), and human (5I17) immunoglobulin structures using multiple structural alignment to assess structural conservation and cross-species antigen recognition (Figure 6).

The three-dimensional structure of the ultralong BOV-2 antibody from *Bos taurus* is shown in Figure 6a, highlighting the heavy chain complementarity-determining region 3 (CDRH3). This region consists of an extended antiparallel β-sheet stalk that supports a globular protruding domain, which is absent in the antibody structures from goat, mouse, and human (Figure 6b–d).

**Figure A2:**
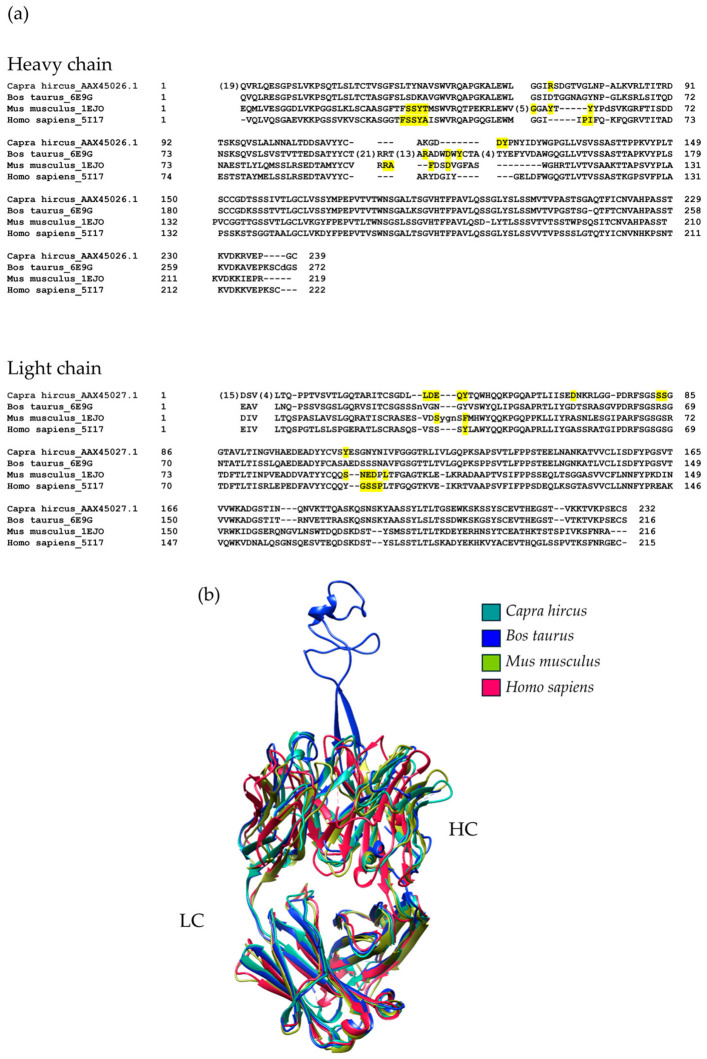
Comparative sequence and structural alignment of mammalian immunoglobulin variable domains. (**a**) Multiple sequence alignment of heavy chain (HC) and light chain (LC) variable regions from *Capra hircus*, *Bos taurus*, *Mus musculus*, and *Homo sapiens* immunoglobulins. The residues that interacted with SRLV p14 nucleocapsid protein in the molecular docking simulation are indicated in yellow. (**b**) Structural superposition of the modeled caprine antibody with experimentally determined bovine (BOV-2, PDB ID: 6E9G), murine (PDB ID: 1EJO), and human (PDB ID: 5I17) immunoglobulin structures.

Despite these differences in the length and topology of antigen-binding loops, structural alignment of the globular variable regions revealed a high degree of interspecies homology. Specifically, the heavy chain of the caprine antibody shared 65% sequence identity with the bovine heavy chain, whereas the light chain exhibited 71% identity with its bovine counterpart (Table 2, Figure A2). In contrast, alignments with murine and human antibodies revealed a more moderate degree of evolutionary conservation, with identity values of approximately 55% for the heavy chain and 44% for the light chain (Table 2, Figure A2).

**Figure 7:**
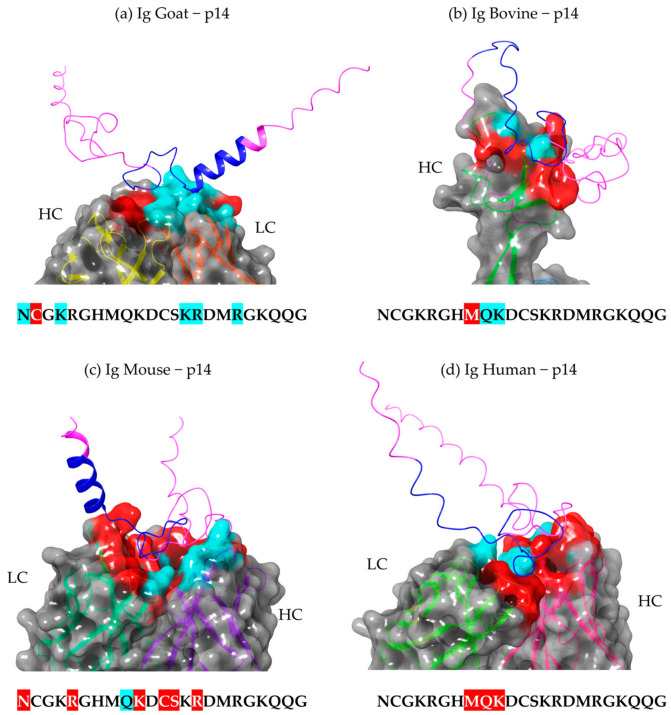
Multi-species molecular docking analysis of the SRLV p14 nucleocapsid protein with caprine, bovine, murine, and human immunoglobulins. Representative docking complexes showcasing structural convergence at the core zinc-finger epitope (region 41–64, highlighted in dark blue) are displayed for the (**a**) AlphaFold-modeled goat antibody, (**b**) bovine ultralong antibody BOV-2, (**c**) mouse antibody 1EJO, and (**d**) human antibody 5I17. Intermolecular interaction networks at the binding interfaces are colored to represent hydrogen bonds (cyan blue) and hydrophobic contacts (red). HC: heavy chain; LC: light chain.

**Figure A3:**
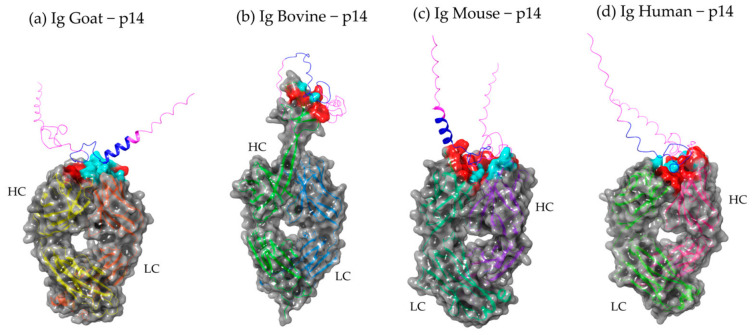
Comparative docking analysis of SRLV p14 nucleocapsid protein with mammalian immunoglobulins. Representative molecular docking complexes obtained using the SRLV p14 nucleocapsid protein as ligand and immunoglobulins from different mammalian species as receptors. (**a**) AlphaFold modeled caprine antibody. (**b**) Bovine ultralong antibody BOV-2 (PDB ID: 6E9G). (**c**) Murine antibody 1EJO (PDB ID: 1EJO). (**d**) Human antibody 5I17 (PDB ID: 5I17). The conserved zinc-finger epitope region of SRLV p14 (residues 41–64) is highlighted. Hydrogen bonds and hydrophobic contacts are represented according to the interaction analysis. HC, heavy chain; LC, light chain.

Regarding the multimeric docking simulations performed in AlphaFold using the SRLV p14 protein, distinct topological preferences were observed among the four mammalian species analyzed (Figure 7, Figure A3).

**Figure A4:**
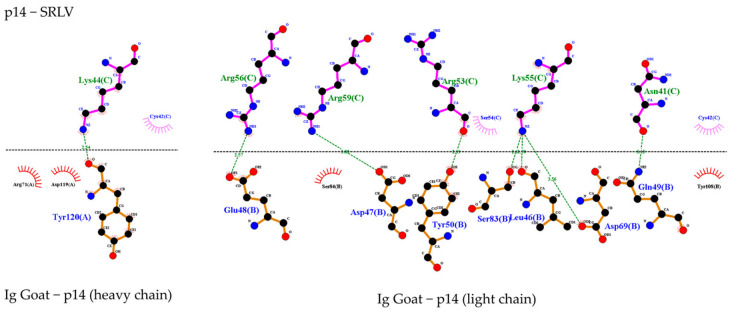
Two-dimensional interaction map of the caprine immunoglobulin-SRLV p14 docking complex. LigPlot representation of intermolecular interactions between the modeled *Capra hircus* immunoglobulin and the SRLV p14 nucleocapsid protein. Hydrogen bonds (green dotted lines) and hydrophobic contacts (red dotted lines) involved in antigen–antibody recognition are indicated.

**Figure A5:**
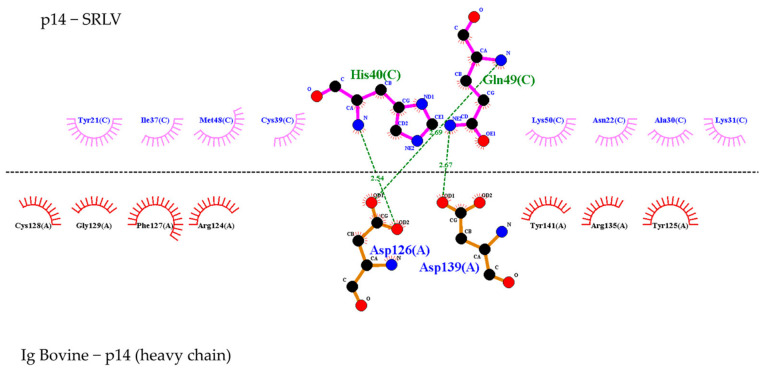
Two-dimensional interaction map of the bovine immunoglobulin-SRLV p14 docking complex. LigPlot representation of intermolecular interactions between antibody bovine (PDB ID: 6E9G; heavy chain) and the SRLV p14 nucleocapsid protein. Hydrogen bonds (green dotted lines) and hydrophobic contacts (red dotted lines) involved in antigen–antibody recognition are indicated.

**Figure A6:**
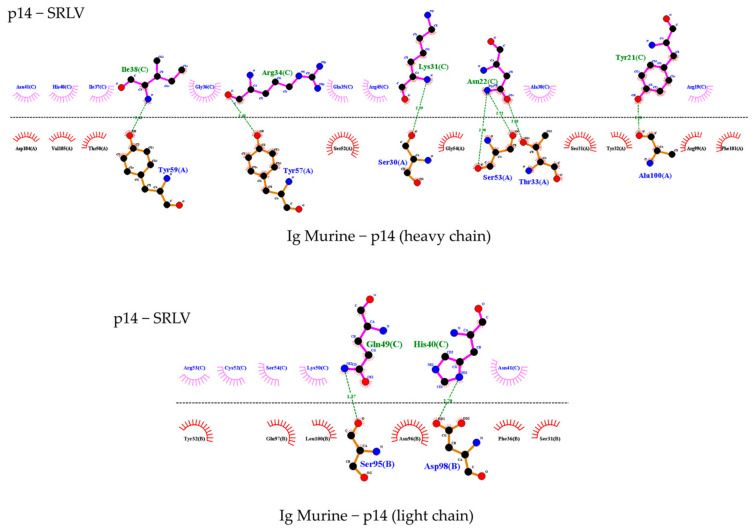
Two-dimensional interaction map of the murine immunoglobulin-SRLV p14 docking complex. LigPlot representation of intermolecular interactions between antibody murine (PDB ID: 1EJO) and the SRLV p14 nucleocapsid protein. Hydrogen bonds (green dotted lines) and hydrophobic contacts (red dotted lines) involved in antigen–antibody recognition are indicated.

**Figure A7:**
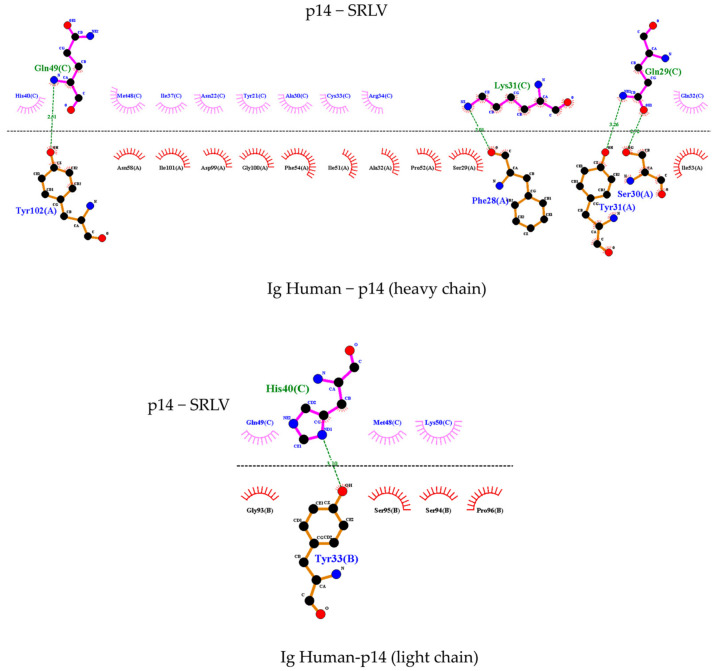
Two-dimensional interaction map of the human immunoglobulin-SRLV p14 docking complex. LigPlot representation of intermolecular interactions between antibody human (PDB ID: 5I17) and the SRLV p14 nucleocapsid protein. Hydrogen bonds (green dotted lines) and hydrophobic contacts (red dotted lines) involved in antigen–antibody recognition are indicated.

Despite the substantial structural variations in the length and conformation of CDR loops among evolutionarily divergent antibody lineages, all four antibody complexes converged on an antigenic region located between residues 41–64 (NCGKRGHMQKDCSKRDMRGKQQG) of the p14 protein (Figure 7). Interface characterization revealed a highly dense and concentrated network of hydrogen bonds (shown in cyan) and hydrophobic contacts (shown in red) distributed along this 23-amino acid linear sequence (Figure 7, Figure A3, Figure A4, Figure A5, Figure A6 and Figure A7). Specifically, residues within the 41–44 region (NCGK) were positioned within the antigen-binding groove of caprine, bovine, murine, and human immunoglobulins (Figure 7). These predictive findings suggest that the spatial arrangement of electrostatic charges and the surface accessibility of this central zinc finger epitope may confer structural compatibility across the mammalian immunoglobulin superfamily.

### 3.7. Presentation of p14 Epitopes by Caprine and Bovine MHC-I Molecules

**Figure A8:**
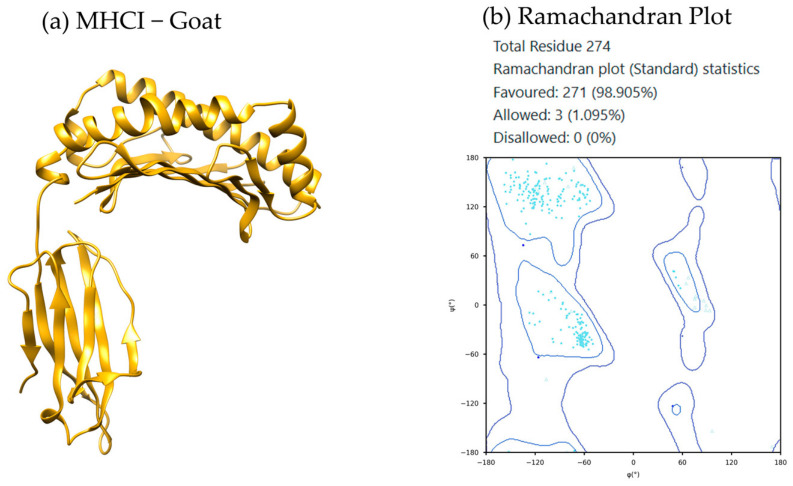
Structural representation and validation of the AlphaFold predicted caprine MHC-I model. (**a**) Three-dimensional model of the *Capra hircus* MHC-I molecule generated using AlphaFold server. (**b**) Ramachandran plot analysis showing residue distribution within favored (light blue dots), allowed (dark blue dots), and disallowed regions (red dots).

**Figure A9:**
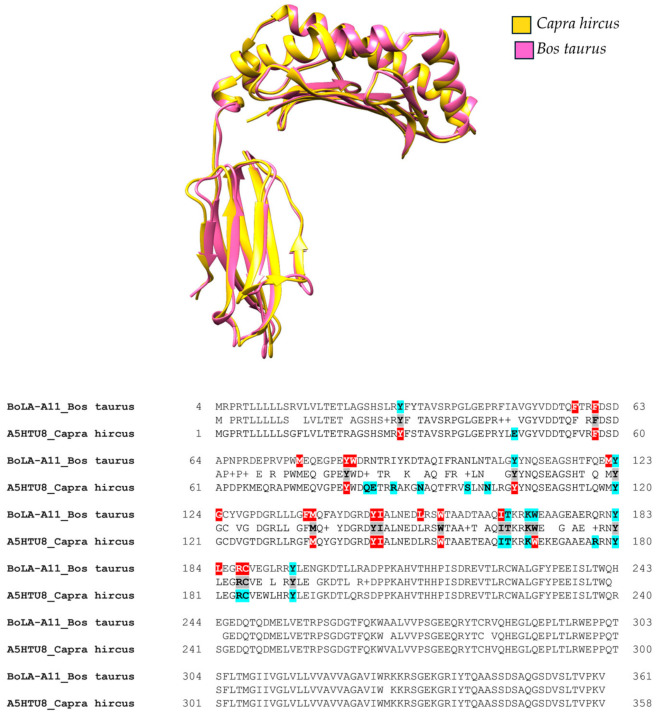
Structural superimposition and sequence alignment of ruminant Major Histocompatibility Complex class I (MHC-I) molecules. The three-dimensional structural overlay shows the high conformational homology between the crystallographic structure of the bovine MHC-I BoLA-A11 (PDB ID: 3PWV, shown in pink) and the AlphaFold predicted model of the caprine MHC-I molecule A5HTU8 (shown in gold). The sequence alignment highlights the conservation of strategic aromatic anchor residues within the longitudinal antigen binding cleft formed between α1 and α2 helices. Intermolecular contact sites identified by docking simulations with SRLV p14 derived antigenic determinants are mapped along the sequences, indicating residues involved in hydrogen bonding (cyan blue) and hydrophobic interactions (red). The alignment of the residues conserved in both proteins is indicated in gray.

Flexible peptide–protein docking simulations performed using the CABS-dock server characterized the integration of five predicted antigenic determinants derived from the SRLV p14 protein into the host cellular antigen presentation machinery. The three-dimensional structure of the caprine MHC-I molecule was modeled using AlphaFold and assessed through structural evaluation, showing 98.90% of residues located within favored regions (Figure A8). To investigate the structural requirements conserved within the ruminant MHC-I superfamily, parallel docking simulations were performed using the experimentally determined structure of the bovine MHC-I molecule BoLA-A11 (PDB ID: 3PWV). Structural superposition and sequence alignment revealed a high degree of identity (85.5%) between caprine and bovine MHC-I receptors (Figure A9).

**Figure 8:**
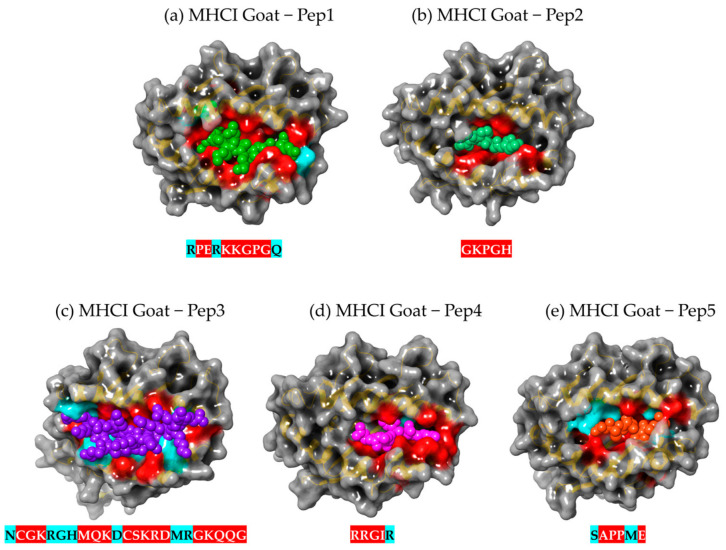
Flexible protein-peptide docking simulations of the five predicted SRLV p14 antigenic determinants within the binding cleft of the caprine MHC-I molecule. Topographical views illustrate the binding modes inside the longitudinal cavity formed between α1 and α2 helices for (**a**) Pep1 (RPERKKGPGQ), (**b**) Pep2 (GKPGH), (**c**) Pep3 (NCGKRGHMQKDCSKRDMRGKQQG), (**d**) Pep4 (RRGIR), and (**e**) Pep5 (SAPPME). The molecular surfaces of the interaction pockets are color coded to delineate functional residues involved in hydrogen bond formations (cyan blue) and stabilized hydrophobic networks (red).

Flexible docking simulations showing that all five p14-derived peptides exhibited affinity for the longitudinal antigen-binding groove formed between the α1 and α2 helices (Figure 8), consistent with the spatial localization of the binding pocket observed in the bovine MHC-I co-crystallized peptide complex [51]. Intermolecular interaction analysis revealed distinct binding modes for each antigenic determinant.

**Figure A10:**
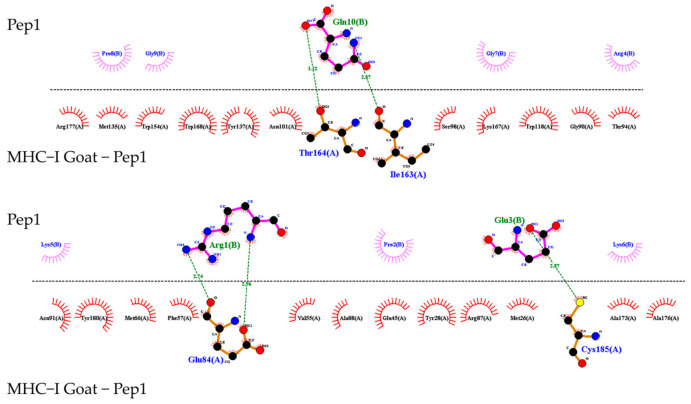
Two-dimensional interaction analysis of SRLV p14-derived peptides (Pep1-RPERKKGPGQ) with caprine MHC-I. LigPlot representation of peptide–MHC-I complexes showing hydrogen bonds (green dotted lines) and hydrophobic contacts (red dotted lines) involved in the stabilization of antigenic peptides within the binding cavity.

**Figure A11:**
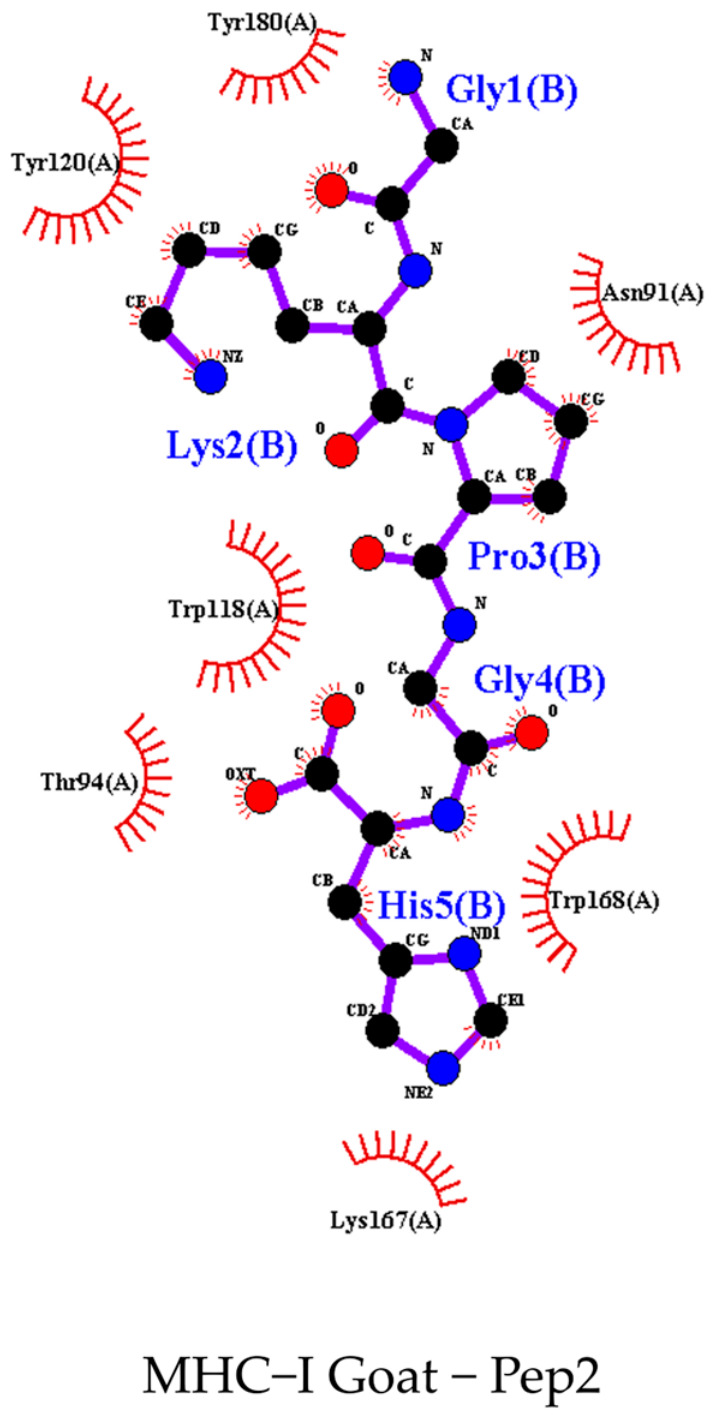
Two-dimensional interaction analysis of SRLV p14-derived peptides (Pep2-GKPGH) with caprine MHC-I. LigPlot representation of peptide–MHC-I complexes showing hydrophobic contacts (red dotted lines) involved in the stabilization of antigenic peptides within the binding cavity.

Pep1 (RPERKKGPGQ) formed three conserved hydrogen bonds with Ile163, Thr164, and Cys185 and established 23 hydrophobic contacts (Figure A10), seven of which involved residues conserved between bovine and caprine receptors (Figure 8a and Figure A9). Pep2 (GKPGH), the shortest ligand evaluated (5 amino acids), was centrally positioned within the binding cavity and stabilized through conserved hydrophobic interactions involving Tyr120, Lys167, Trp168, and Tyr180 (Figure 8b, Figure A9 and Figure A11).

**Figure A12:**
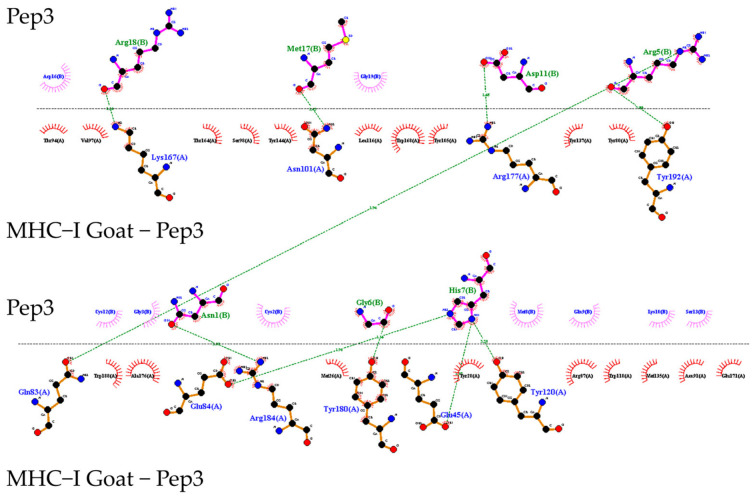
Two-dimensional interaction analysis of SRLV p14-derived peptides (Pep3-NCGKRGHMQKDCSKRDMRGKQQG) with caprine MHC-I. LigPlot representation of peptide–MHC-I complexes showing hydrogen bonds (green dotted lines) and hydrophobic contacts (red dotted lines) involved in the stabilization of antigenic peptides within the binding cavity.

Pep3 (NCGKRGHMQKDCSKRDMRGKQQG), the longest predicted antigenic determinant (23 amino acids), established the most extensive and complex network of physical contacts across the entire peptide-binding groove (Figure 8c). Structural stabilization was mediated by five hydrogen bonds involving the conserved residues Tyr120, Lys167, Tyr180, Arg184, and Tyr192 (Figure 8c, Figure A9 and Figure A12), together with a dense hydrophobic interaction network involving conserved residues Tyr28, Tyr80, Tyr105, Met135, Tyr144, Thr164, and Trp168 (Figure 8c, Figure A9 and Figure A12).

**Figure A13:**
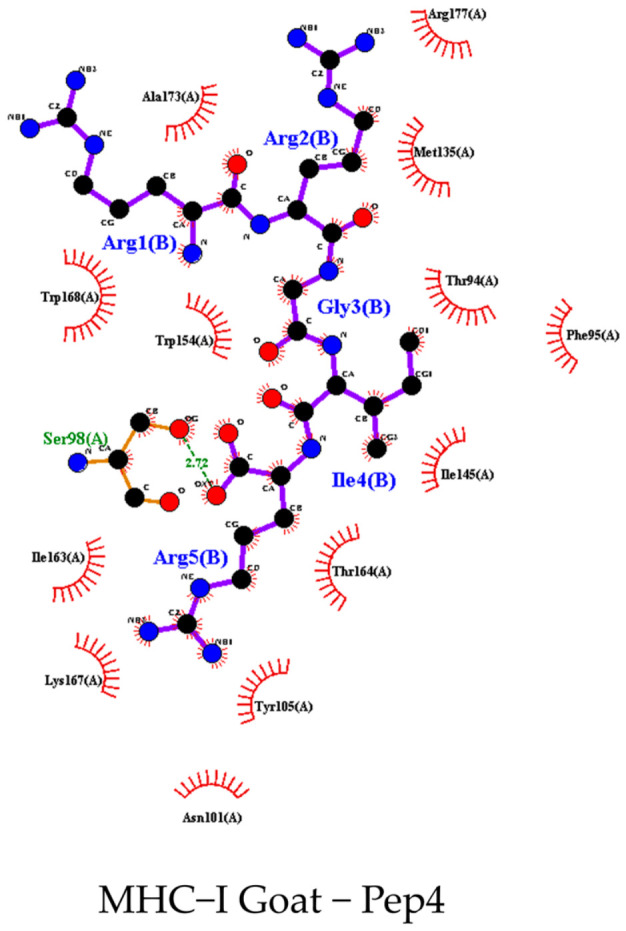
Two-dimensional interaction analysis of SRLV p14-derived peptides (Pep4-RRGIR) with caprine MHC-I. LigPlot representation of peptide–MHC-I complexes showing hydrogen bonds (green dotted lines) and hydrophobic contacts (red dotted lines) involved in the stabilization of antigenic peptides within the binding cavity.

**Figure A14:**
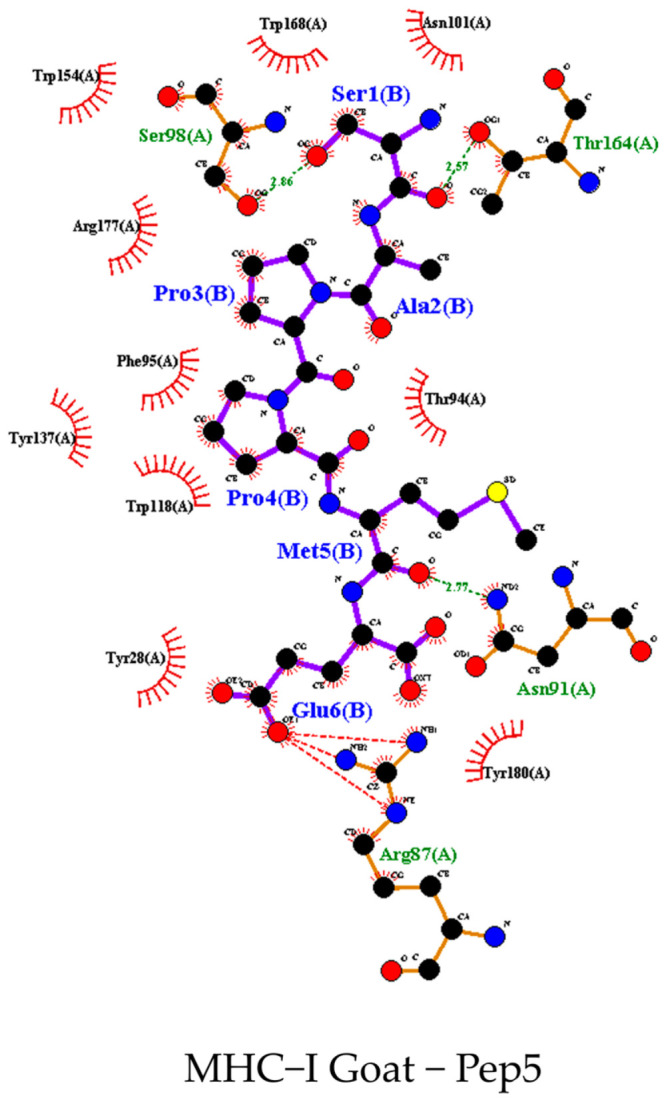
Two-dimensional interaction analysis of SRLV p14-derived peptides (Pep5-SAPPME) with caprine MHC-I. LigPlot representation of peptide–MHC-I complexes showing hydrogen bonds (green dotted lines) and hydrophobic contacts (red dotted lines) involved in the stabilization of antigenic peptides within the binding cavity.

For Pep4 (RRGIR) and Pep5 (SAPPME), both peptides exhibited favorable positioning within the antigen-binding groove (Figure 8d and Figure 8e respectively). These interactions included a key hydrogen bond with the conserved residue Thr164 (Pep5), complemented by hydrophobic contacts involving Phe95, Trp158, and Trp168 (Pep4 and Pep5) (Figure A9, Figure A13 and Figure A14).

## 4. Discussion

The primary objective of this study was not to elucidate the biological functions of the p14 protein, but rather to perform an in-depth structural and antigenic characterization. The SRLV, HIV-1, FIV, and EIAV lentivirus nucleoproteins had a more than 40% homology between the proteins in three-dimensional modeling; however, the shared “horseshoe”-type structure, corresponding to the zinc fingers characteristic of proteins that bind nucleic acids such as nucleoproteins, is sufficient, alongside similar percentages of identity in the three-dimensional model and putative distributions of antigenic determinants Prediction of antigenic determinants in the nucleoprotein sequences revealed that p14 protein of SRLV contains several hydrophilic and antigenic regions distributed throughout its amino acid sequence. Notably, the epitopes predicted by the ElliPro server were predominantly located within these regions, indicating a strong concordance between the computational predictions and the physicochemical properties of the protein. These findings suggest that the identified epitopes may contribute substantially to the antigenic profile of p14. These findings provide further evidence supporting the antigenic potential of SRLV nucleoprotein and underscore its relevance as a candidate target for immunological applications.

The lentiviruses SRLV, HIV-1, FIV, and EIAV share a specific and functional “horseshoe” structure that corresponds to the zinc-finger domains responsible for the interaction of nucleoproteins with nucleic acid molecules. The lentiviruses’ sequence is Cys-X2-Cys-X4-His-X4-Cys, where “X” represents any amino acid that can vary between different retroviruses and are connected by a basic linker sequence of variable length [52,53,54,55,56,57,58]. They are also connected by a basic linker sequence of variable length [59]. This structure exhibited greater similarity in the nucleoproteins of the SRLV and EIAV viruses. Prediction of antigenic determinants in the nucleoprotein sequences suggests that most of the epitopes found were structural epitopes. Furthermore, the nucleoproteins of SRLV, HIV-1, and FIV shared epitopes at the end of the sequence, suggesting that this is likely a structurally conserved region among these lentiviruses and may play a relevant role in the antigenic properties of the protein, although genetic studies are required to confirm this.

The correct cloning of the SRLV-rp14 nucleoprotein of genotype B in the pETSUMO expression plasmid was achieved and corroborated using Western blot, PCR, and sequencing. The recombinant protein with a molecular weight of 26 KDa was efficiently expressed due to the tags added at amino terminal end by cloning the coding sequence of p14 with pETSUMO plasmid. The recombinant protein was efficiently produced and successfully purified, as observed in the elutions obtained via affinity column purification, SDS-PAGE analysis, and Western blot and the integrity have been reported in other studies that used solid phase immunodetection [8]. The lower-intensity bands detected may be associated with post-translational modifications or protein processing events occurring during expression in BL21-CAT strain of Escherichia coli. Nevertheless, the recombinant protein was purified to an estimated purity of 96%.

Antigenicity was confirmed via Western blot using 300 ng of SRLV-rp14. The results for the presented immunoreactivity were obtained using the plasmas of sheep and goats from several states of Mexico naturally infected with SLRV. In previous work, same plasma samples were previously analyzed using 500 ng of SRLV-rp25 protein of the purified protein confirming antigenicity of recombinant proteins in a heterologous system [10]. In this work, 78.7% of samples presented immunoreactivity toward the nucleocapsid protein, thus suggesting the antigenic capacity of the protein to be recognized by antibodies produced by animals naturally infected with SRLV. These results agree with the reported immunoreactivity range of 69–88% for Gag proteins (p14, p17, and p25), although the authors did not report the individual percentage for each protein analyzed [56]. Although this was not an epidemiological study.

Currently, SRLV infection is detected using diagnostic test kits and PCR, and the results could be corroborated using Western blot. The negative results for the samples analyzed do not necessarily indicate non-infected animals; this may be due to the antibody titer being below the detection level of the recombinant protein [8]. The difference in the degree of recognition toward SRLV-rp14 presented in the samples analyzed could be due to the antibody titer produced against this protein, which depends on the stage of infection and each animal’s response [40,59,60]. However, our results demonstrated a concordance among the samples analyzed, despite differences in both detection methodologies and antigenic targets employed by respective assays. Based on the results obtained, it suggests in vivo assays must be conducted using adjuvants that increase the immune response to determine the immunogenic capacity. Furthermore, it is necessary to investigate its use as a diagnostic system using recombinant proteins.

The p25 protein has been studied for use in diagnostic and immunization systems [10,12]. Nevertheless, it is necessary to improve the analysis of antigenicity and structure of other viral proteins, such as the recombinant protein SRLV-rp14, which in our results also shows antigenicity capacity. Considering there is not a “gold standard” test for diagnostic o SRLV infection is necessary to keep investigating antigenic and structural properties of SRLV proteins.

The integration of bioinformatic approaches allowed us to address a major limitation in the study of small ruminant immunology: the lack of experimentally resolved structures specific to these species. Using AlphaFold, three-dimensional models of caprine immunoglobulins and major histocompatibility complex class I (MHC-I) molecules were generated, providing a molecular framework based on host-specific structures and avoiding exclusive reliance on crystallographic models obtained from phylogenetically related species.

A relevant finding from our multispecies in silico analysis was the identification of a convergent antigen recognition pattern among caprine, bovine, murine, and human immunoglobulins toward the linear epitope of 23 amino acids (Pep3: NCGKRGHMQKDCSKRD-MRGKQQG), located within the zinc-finger region of the SRLV p14 nucleocapsid protein. Despite structural differences among immunoglobulins from these species, docking models predicted a conserved interaction with this viral region. This suggests that recognition of Pep3 may be primarily influenced by epitope accessibility, surface charge distribution, and spatial organization rather than species-specific antibody architecture. This observation is relevant considering the evolutionary differences in antibody repertoires among ruminants. Cattle possess an exceptional immunoglobulin repertoire characterized by long CDRH3 regions, which enable recognition of structurally complex antigens through extended binding surfaces [43,61]. In contrast, goats and sheep exhibit shorter CDRH3 regions, more similar to those described in mice and humans [62,63]. Therefore, the convergence observed among these species toward a similar structural region of Pep3, particularly near the NCGK motif, suggests that this region of p14 contains intrinsic structural properties favorable for immune recognition.

The SRLV p14 nucleocapsid protein has essential roles beyond RNA binding, including genome packaging, dimerization, and regulation of viral replication processes [64]. Like other retroviral nucleocapsid proteins, such as HIV-1 [65,66], p14 contains conserved zinc-finger motifs involved in ribonucleoprotein organization and interactions with host factors. In our models, the SRLV zinc-finger region exhibited structural characteristics compatible with a dynamic domain, combining defined secondary structures with flexible regions. This conformational plasticity may contribute to epitope exposure and facilitate immune recognition.

The immunological relevance of Pep3 was further supported by antigen presentation analysis using CABS-dock simulations. The predicted caprine Pep3-MHC-I complex showed a spatial arrangement comparable to the experimentally resolved bovine BoLA-A11 peptide complex. The conservation of residues involved in peptide-binding pockets suggests shared structural constraints among ruminant MHC-I molecules [67]. Particularly, the predicted interaction with conserved anchoring residues provides a potential explanation for the presentation of viral peptides derived from internal proteins and their recognition by cytotoxic T lymphocytes [51,68].

The identification of viral regions capable of inducing both humoral and cellular immune responses is particularly relevant considering the high evolutionary plasticity of SRLVs [69]. These viruses represent a continuum between caprine arthritis encephalitis (CAEV) virus and maedi-visna virus (MVV) variants [70], with evidence of interspecies transmission, recombination events, and adaptation to new hosts [71,72]. The high mutation rate associated with reverse transcriptase activity generates heterogeneous viral populations under strong immune selection pressure [35]. Therefore, structural characterization of p14-derived peptides and their interaction with caprine MHC-I molecules provides insight into how viral variability may affect immune recognition by considering not only sequence conservation but also structural accessibility, stability, and compatibility with antigen presentation pathways.

The relevance of conserved antigenic regions has also been demonstrated in SRLV diagnostics. Previous studies have shown that ELISA performance varies depending on viral genotype and antigen composition, indicating that no single assay can universally detect all infections [73]. The use of synthetic peptides derived from conserved viral regions has improved genotype discrimination, emphasizing the importance of selecting functionally relevant epitopes for diagnostic and vaccine development [73].

Consequently, the approach developed in this study, combining AlphaFold-based structural prediction, immune complex modeling, and peptide–MHC-I interaction analysis, represents a complementary approach for identifying immunologically relevant viral regions. Incorporating structural information beyond sequence-based analyses enables the selection of candidate epitopes according to their three-dimensional organization, accessibility, and interaction potential with host immune molecules.

Nevertheless, several limitations should be considered. AlphaFold models represent predicted static conformations and may not fully reproduce protein flexibility under physiological conditions [74]. Likewise, molecular docking results indicate structural compatibility rather than direct measurements of binding affinity. Another important limitation concerns the prediction of antigenic epitopes using ElliPro and their subsequent evaluation via molecular docking in CABS-dock. ElliPro predicts putative B-cell epitopes based primarily on protein geometry, solvent accessibility, and protrusion index rather than on experimentally determined antigenicity [32]. Consequently, the identified epitopes should be interpreted as structural candidates rather than confirmed immunogenic determinants. Furthermore, docking of these predicted peptides to MHC-I molecules evaluates structural compatibility within the peptide-binding groove but does not reproduce the complete antigen-processing pathway, which involves proteasomal cleavage, TAP-mediated transport, peptide editing, MHC loading, and T-cell receptor recognition. Therefore, the docking results should be interpreted as evidence supporting the structural feasibility of peptide presentation rather than proof of naturally processed or immunodominant CD8^+^ T-cell epitopes [51,67]. Validation through complementary bioinformatics approaches and experimental methods, including antibody-binding assays, synthetic peptide mapping, and T-cell functional studies, will be necessary to establish the biological relevance of the predicted epitopes.

Finally, SRLV genetic diversity remains an important challenge. Although p14 contains conserved immunogenic regions, viral evolution may generate substitutions affecting antibody recognition or antigen presentation. The present analysis was based on representative sequences and did not encompass the full diversity of circulating variants. Future studies integrating larger genomic datasets from diverse geographical regions and host species will be necessary to evaluate Pep3 conservation and its potential as a broadly applicable diagnostic or vaccine target.

## 5. Conclusions

This study analyzed, for the first time, the antigenic capacity of the SRLV-rp14 nucleoprotein of genotype B1, which mainly circulates in Mexico. A larger sample of sheep and goats was used compared to those reported by authors from other countries. The three-dimensional structure of the SRLV p14 protein was analyzed and compared with the lentivirus nucleoproteins with the highest similarity. Structural epitopes were found in the zinc-finger structures and epitopes that could be conserved in the nucleoproteins of the SRLV, HIV-1, and FIV retroviruses. The antigenic capacity observed in SRLV-rp14 suggests that this protein is an ideal immunogenic candidate to be studied in combination with adjuvants in new-generation vaccines. Furthermore, multispecies molecular docking simulations revealed structural convergence across species. Despite structural variations among mammalian lineages, caprine, bovine, murine, and human immunoglobulins all targeted a 23-amino-acid region (residues 41–64) located within the central zinc finger domain. Lastly, flexible peptide–protein docking simulations using the CABS-dock platform predict that linear antigenic determinants derived from p14 exhibit steric compatibility with the longitudinal antigen-presenting cleft of both caprine and bovine MHC-I, supporting their candidacy for further experimental validation rather than demonstrating physiological antigen presentation. These findings position SRLV-rp14 as a promising, immunologically conserved target for next-generation multi-epitope diagnostics and subunit vaccines against small ruminant lentiviruses, although experimental validation is still required to definitively confirm their biological functionality.

## Figures and Tables

**Table 1:** Predicted antigenic determinants of SRLV, EIAV, FIV, and HIV-1 Lentiviral nucleoproteins identified using the ElliPro server.

Lentivirus	Number	Start	End	Residues Number	Sequence
SRLV	1	7	46	10	RPERKKGPGQ
	2	24	28	5	GKPGH
	3	41	64	24	NCGKRGHMQKDCSKRDMRGKQQG
	4	67	71	5	RRGIR
	5	75	80	6	SAPPME
VAIE	1	27	38	12	KQPGHFSKQCRS
VIF	1	1	5	5	PGQLF
	2	12	26	15	KPGHMSRQCRAPRKC
	3	28	35	8	NCGKTGHI
	4	38	48	11	DCWQMKGKQQG
	5	51	57	7	QQGRAAA
	6	72	78	7	SLQQTAP
	7	82	87	6	SAPPME
VIH-1	1	1	5	5	KGPRR
	2	13	17	5	GKEGH
	3	24	29	6	APREKG
	4	32	48	17	KCGKEGHQMKDCTERQA
	5	56	62	7	PSRQGRP
	6	66	79	14	IQTTRLEPTAPPAE

**Table 2:** Sequence identity percentages of bovine, murine, and human immunoglobulins are relative to the AlphaFold modeled caprine antibody. Comparative alignment analysis was performed independently for the heavy and light chain domains of each species against the caprine reference host model.

Chain	Identity Percentage		
	6E9G Ig Bovine	1EJO Ig Mouse	5I17 Ig Human
Heavy chain	65%	54%	55%
Light chain	71%	44%	43%

## Data Availability

Dataset available on request from the authors.

## Abbreviations

The following abbreviations are used in this manuscript:

SRLV	Small ruminant lentivirus
MHC-I	Major Histocompatibility Complex

## Appendix A

**Table A1:** Immunoreactivity analysis degree of SRLV-rp14 by Western blot in previously evaluated serum by commercial Kits for Laboratory Diagnosis of SRLV (VMRD-gp135 and ERADIKIT-Peptides Gag). A high degree of recognition is indicated by four marks (++++), a moderate degree by three (+++), a medium degree by two (++), and a low degree by one (+); the negative control serum is indicated by a single mark (-).

SRLV-rpP14					
Sample	Specie	State of Mexico	VMRD Gp135	ERADIKIT Peptides Gag	Western-Blot Degree of Recognition
1	Goat	Estado de México	POSITIVE	POSITIVE	++++
2	Goat	Guanajuato	POSITIVE	POSITIVE	++++
3	Goat	Sinaloa	POSITIVE	POSITIVE	++++
4	Goat	Guanajuato	POSITIVE	POSITIVE	++++
5	Goat	Veracruz	POSITIVE	POSITIVE	++++
6	Goat	Veracruz	NEGATIVE	NEGATIVE	++++
7	Goat	ND	ND	ND	++++
8	Goat	ND	ND	ND	++++
9	Goat	Baja California Sur	POSITIVE	POSITIVE	+++
10	Goat	Sonora	POSITIVE	POSITIVE	+++
11	Goat	Sinaloa	POSITIVE	POSITIVE	+++
12	Goat	Veracruz	POSITIVE	POSITIVE	+++
13	Goat	Guanajuato	NEGATIVE	NEGATIVE	+++
14	Goat	Guanajuato	NEGATIVE	NEGATIVE	+++
15	Goat	Sinaloa	POSITIVE	POSITIVE	+++
16	Goat	Tlaxcala	NEGATIVE	NEGATIVE	+++
17	Goat	Sonora	POSITIVE	POSITIVE	+++
18	Goat	ND	ND	ND	+++
19	Goat	ND	ND	ND	+++
20	Goat	ND	ND	ND	+++
21	Goat	ND	ND	ND	+++
22	Goat	ND	ND	ND	+++
23	Goat	Sinaloa	POSITIVE	POSITIVE	++
24	Goat	Sinaloa	POSITIVE	POSITIVE	++
25	Goat	Sinaloa	NEGATIVE	NEGATIVE	++
26	Goat	Estado de México	POSITIVE	POSITIVE	++
27	Goat	Guanajuato	POSITIVE	POSITIVE	++
28	Goat	Guanajuato	NEGATIVE	NEGATIVE	++
29	Goat	Veracruz	NEGATIVE	NEGATIVE	++
30	Goat	Sinaloa	NEGATIVE	NEGATIVE	++
31	Goat	Guanajuato	NEGATIVE	NEGATIVE	++
32	Goat	Guanajuato	NEGATIVE	NEGATIVE	++
33	Goat	Veracruz	NEGATIVE	NEGATIVE	++
34	Goat	Guanajuato	POSITIVE	POSITIVE	++
35	Goat	Querétaro	NEGATIVE	NEGATIVE	++
26	Sheep	Hidalgo	NEGATIVE	NEGATIVE	++
37	Goat	Coahuila	NEGATIVE	NEGATIVE	++
38	Goat	Veracruz	NEGATIVE	NEGATIVE	++
39	Goat	Sinaloa	POSITIVE	POSITIVE	++
40	Goat	Veracruz	NEGATIVE	NEGATIVE	++
41	Goat	ND	ND	ND	++
42	Goat	ND	ND	ND	++
43	Goat	ND	ND	ND	++
44	Goat	Guanajuato	POSITIVE	POSITIVE	++
45	Goat	ND	ND	ND	++
46	Goat	Estado de México	POSITIVE	POSITIVE	++
47	Goat	Guanajuato	POSITIVE	POSITIVE	+
48	Goat	Coahuila	POSITIVE	POSITIVE	+
49	Goat	Coahuila	NEGATIVE	NEGATIVE	+
50	Goat	Coahuila	NEGATIVE	NEGATIVE	+
51	Goat	Coahuila	NEGATIVE	NEGATIVE	+
52	Sheep	Hidalgo	NEGATIVE	NEGATIVE	+
53	Goat	Veracruz	NEGATIVE	NEGATIVE	+
54	Goat	Veracruz	NEGATIVE	NEGATIVE	+
55	Goat	Veracruz	NEGATIVE	NEGATIVE	+
56	Goat	ND	ND	ND	+
57	Goat	ND	ND	ND	+
58	Goat	ND	ND	ND	+
59	Goat	ND	ND	ND	+
60	Goat	ND	ND	ND	+
61	Goat	ND	ND	ND	+
62	Goat	Veracruz	NEGATIVE	NEGATIVE	-
63	Goat	Querétaro	NEGATIVE	NEGATIVE	-
64	Goat	Sinaloa	NEGATIVE	NEGATIVE	-
65	Sheep	Estado de México	NEGATIVE	NEGATIVE	-
66	Goat	Guanajuato	POSITIVE	POSITIVE	-
67	Sheep	Estado de México	POSITIVE	POSITIVE	-
68	Goat	Sinaloa	NEGATIVE	NEGATIVE	-
69	Sheep	Baja California Sur	NEGATIVE	NEGATIVE	-
70	Goat	Durango	NEGATIVE	NEGATIVE	-
71	Goat	Sinaloa	NEGATIVE	NEGATIVE	-
72	Goat	Veracruz	NEGATIVE	NEGATIVE	-
73	Goat	Durango	NEGATIVE	NEGATIVE	-
74	Sheep	Estado de México	NEGATIVE	NEGATIVE	-
75	Sheep	ND	ND	ND	-
76	Sheep	ND	ND	ND	-
77	Sheep	ND	ND	ND	-
78	Sheep	ND	ND	ND	-
79	Sheep	ND	ND	ND	-

ND means not determined.
